# Embryonic development and perinatal skeleton in a limbless, viviparous lizard, *Anguis fragilis* (Squamata: Anguimorpha)

**DOI:** 10.7717/peerj.11621

**Published:** 2021-06-17

**Authors:** Tomasz Skawiński, Grzegorz Skórzewski, Bartosz Borczyk

**Affiliations:** 1 Department of Evolutionary Biology and Conservation of Vertebrates, University of Wroclaw, Wrocław, Poland; 2 Museum of Natural History, University of Wroclaw, Wrocław, Poland

**Keywords:** Evo-devo, Morphology, Neonates, Osteology, Squamates, Variation

## Abstract

Despite the long history of embryological studies of squamates, many groups of this huge clade have received only limited attention. One such understudied group is the anguimorphs, a clade comprising morphologically and ecologically very diverse lizards. We describe several stages of embryonic development of *Anguis fragilis*, a limbless, viviparous anguimorph. Interestingly, in several clutches we observe high morphological variation in characters traditionally important in classifying embryos into developmental stages. The causes of this variation remain unknown but environmental factors do not seem to be very important. Additionally, we describe the state of ossification in several perinatal specimens of *A. fragilis*. The cranial skeleton is relatively poorly ossified around the time of birth, with all of the bones constituting the braincase unfused. On the other hand, the vertebral column is well ossified, with the neurocentral sutures closed and the neural arches fused in all postatlantal vertebrae. Such an advanced state of ossification may be related to the greater importance of the vertebral column in locomotion in limbless species than in ones with fully-developed limbs. Numerous factors seem to affect the state of ossification at the time of hatching or birth in squamates, including phylogenetic position, mode of reproduction and, potentially, limblessness. However, data from a greater number of species are needed to reach firmer conclusions about the relative importance of these variables in certain clades.

## Introduction

Squamates include over 11,000 living species (Uetz, Freed & Hošek, 2021), which makes them one of the largest clades of extant vertebrates. They live in different environments and show various modifications of their basic body plan, including but not limited to different degrees of limb reduction. The origins of this diversity may be illuminated by studies on embryonic development. The development of squamates has been studied for a long time (e.g.,  Leydig, 1872; De Beer, 1930) and there are numerous publications on the subject. For example, developmental staging tables (some complete, others restricted to certain developmental period or body structure) are available for more than 60 species (see Ollonen et al., 2018). However, some important squamate clades received only a little attention in embryological studies. Anguimorpha, although not particularly species-rich (the group comprises approximately 240 currently accepted extant species; Uetz, Freed & Hošek, 2021), are one of the major squamate clades and include morphologically and ecologically very diverse species; limbless burrowers (e.g., *Anniella pulchra*), terrestrial species with fully-developed limbs (some anguids, xenosaurids, helodermas, many species of *Varanus*), and even semiaquatic lizards (some species of *Varanus*, *Shinisaurus crocodilurus* and *Lanthanotus borneensis*) (Pianka & Vitt, 2003). Complete developmental staging tables are available only for three species of monitor lizards (Gregorovicova et al., 2012; Werneburg, Polachowski & Hutchinson, 2015; Andrews et al., 2017) and cranial development of *Elgaria coerulea* (Good, 1995). Such limited data obscure the true variation present within Anguimorpha and hinder large-scale evolutionary analyses of squamate development (e.g.,  Maisano, 2002; Andrews, Brandley & Greene, 2013; Werneburg & Sánchez-Villagra, 2015; Skawiński & Borczyk, 2017).

To fill part of this gap, we studied the development of the slow worm (*Anguis fragilis*), a species of medium-sized, limbless, viviparous anguimorph lizard, widely distributed in Europe (e.g.,  Gvoždík et al., 2010; Gvoždík et al., 2013). Even though its early development, from the cleavage to the closure of the amnion, has been thoroughly studied (Nicolas, 1904; Ballowitz, 1905; Meyer, 1910), as well as the genital (Raynaud, 1963) and limb bud development (see review in Raynaud, 1985), only limited attention has been paid to later stages (e.g.,  Raynaud, 1959). Therefore, many aspects of development in slow worms are still poorly known. Several papers dealt with (or at least commented on) skeletal development but focused only on selected structures or regions such as integument (Maderson, 1965), vertebrae (Winchester & Bellairs, 1977), sacral region (Borkhvardt & Malashichev, 2000) or skull, including the chondrocranium (Leydig, 1872; Zimmermann, 1913; Yaryhin et al., 2021^1^), 1Based on the geographic origin of their sample (eastern Slovakia), it seems that Yaryhin et al. (2021) studied most probably *Anguis colchica* which used to be regarded as a subspecies of *A. fragilis* but is now considered a distinct, although very closely related, species (e.g., Gvoždík et al., 2010; Speybroeck et al., 2020).ethmoidal (Pratt, 1948), orbitotemporal (Bellairs, 1949) or frontoparietal (Da Silva et al., 2018) regions. We here supplement these works by providing descriptions of embryo morphology at several stages, as well as perinatal and early postnatal osteology. Information about the neonatal skeleton in squamates are scarce. This is especially true for anguimorphs, for which only data for viviparous anguid *Elgaria coerulea* are available (Maisano, 2001). Its skeleton was poorly ossified and we predict that the degree of skeletal development is similar in perinatal slow worms.

## Material & Methods

This study is based on five perinatal slow worms and 42 embryos dissected out of 7–9 ethanol-preserved females (the mother is not known in three embryos but they are likely from the same clutch; Table 1, Table S1). These specimens are in collections of the Museum of Natural History (MNHW) or the Department of Evolutionary Biology and Conservation of Vertebrates (IZK), both at the University of Wrocław. The females were identified as *Anguis fragilis* based on morphology (Sos, 2011), and locality when known (all were collected in areas where *A. fragilis* is the only species of *Anguis*; e.g., Jablonski et al., 2017; Table 1). The majority of these females (presumably all) were collected in the field, so the exact age of the embryos can only be approximated based on the date of collection of the mother (Table 1). We assessed the developmental stage of the embryos based on the examination of their morphology under Zeiss Stemi SV 11 stereomicroscope, with particular reference to characters from the Standard Event System (Werneburg, 2009) and those commonly used in developmental tables of squamate embryos. Photographs were taken using Zeiss AxioCam HRc camera mounted on a stereomicroscope. As the embryos are very fragile and many of them were strongly curled, we made all measurements using images. However, because not all developmental stages are represented in our sample, the descriptions below are not equivalent to classical staging tables. For comparison, we used several skeletonised adult and juvenile slow worms (IZK 01004, IZK 01005, IZK 01006) and one double-stained adult specimen (MNHW-Reptilia-280).

**Table 1:** Basic information about analysed embryos of *Anguis fragilis*.

Female (catalogue number)	Snout-vent length	Date of collection	Collection site and coordinates	Number of embryos examined
MNHW-Reptilia-0251	152 mm	ca. July-August 2015	Pszczew 52.478 N, 15.780 E	8
MNHW-Reptilia-0316-3	143 mm	July 2016	Pszczew 52.478 N, 15.780 E	4
MNHW-Reptilia-0315-4	163 mm	9 July 2015	Bartniki 52.011 N, 20.250 E	2
MNHW-Reptilia-0315-5	177 mm	9 July 2015	Bartniki 52.011 N, 20.250 E	6
IZK 01007	Damaged specimen	Unknown	Unknown, presumably SW Poland	8
IZK 01008	Damaged specimen	Unknown	Unknown, presumably SW Poland	11
–	Unknown	Unknown	Unknown	3

The snout-vent length (SVL) of newly born slow worms varies between 40 and 56 mm (48–49 mm on average), based on data from northwestern Spain (Ferreiro & Galán, 2004). Because it was usually unknown whether the specimens in our sample were dissected out of captured females or collected after birth, we refer to them collectively as ’perinatal’ (only the largest specimen, MNHW-Reptilia-0312, with SVL = 55 mm, was collected definitely after birth, and the second-largest specimen, MNHW-Reptilia-0311-1, with SVL = 49 mm, was collected most probably after birth). The perinatal slow worms and a few embryos were double-stained (following the method described by Dingerkus & Uhler, 1977 with slight modifications) to analyse their skeletal development. The specimens were stained with alcian blue for demonstration of cartilage, digested in a sodium borate-pancreatin solution and subsequently stained with alizarin red S to visualise the calcifications. Subsequently, the specimens were transferred through a series of KOH-glycerin solutions and ultimately stored in pure glycerin with the addition of thymol (Dingerkus & Uhler, 1977). In the four smallest perinatal specimens (SVL between 44 and 49 mm), which were collected in the 1960s, the alcian blue did not bond to the cartilage, so the descriptions are based entirely on the alizarin-stained calcifications.

Complete osteological description of the perinatal skeleton is beyond the scope of the present article (although we acknowledge that such work is desirable in light of recent taxonomic revisions of *Anguis* –Gvoždík et al., 2010; Gvoždík et al., 2013). However, because of the paucity of data on the osteology of slow worms and anguines in general (Rieppel, 1980; Klembara et al., 2017; Villa & Delfino, 2019), we provide a brief description of the “external osteology” of articulated skulls of double-stained specimens. Descriptions of bone morphology are based primarily on the largest and most mature individual (MNHW-Reptilia-0312), while descriptions of ontogenetic changes are based on all double-stained specimens. We put particular emphasis on characters distinguished by Maisano (2001) and known to show interspecific differences in squamates. Identification and nomenclature of bones and other anatomical structures follow the works of Maisano (2001), Evans (2008), Klembara et al. (2017) and Villa & Delfino (2019).

## Results

### Morphology of the embryos

**Figure 1:**
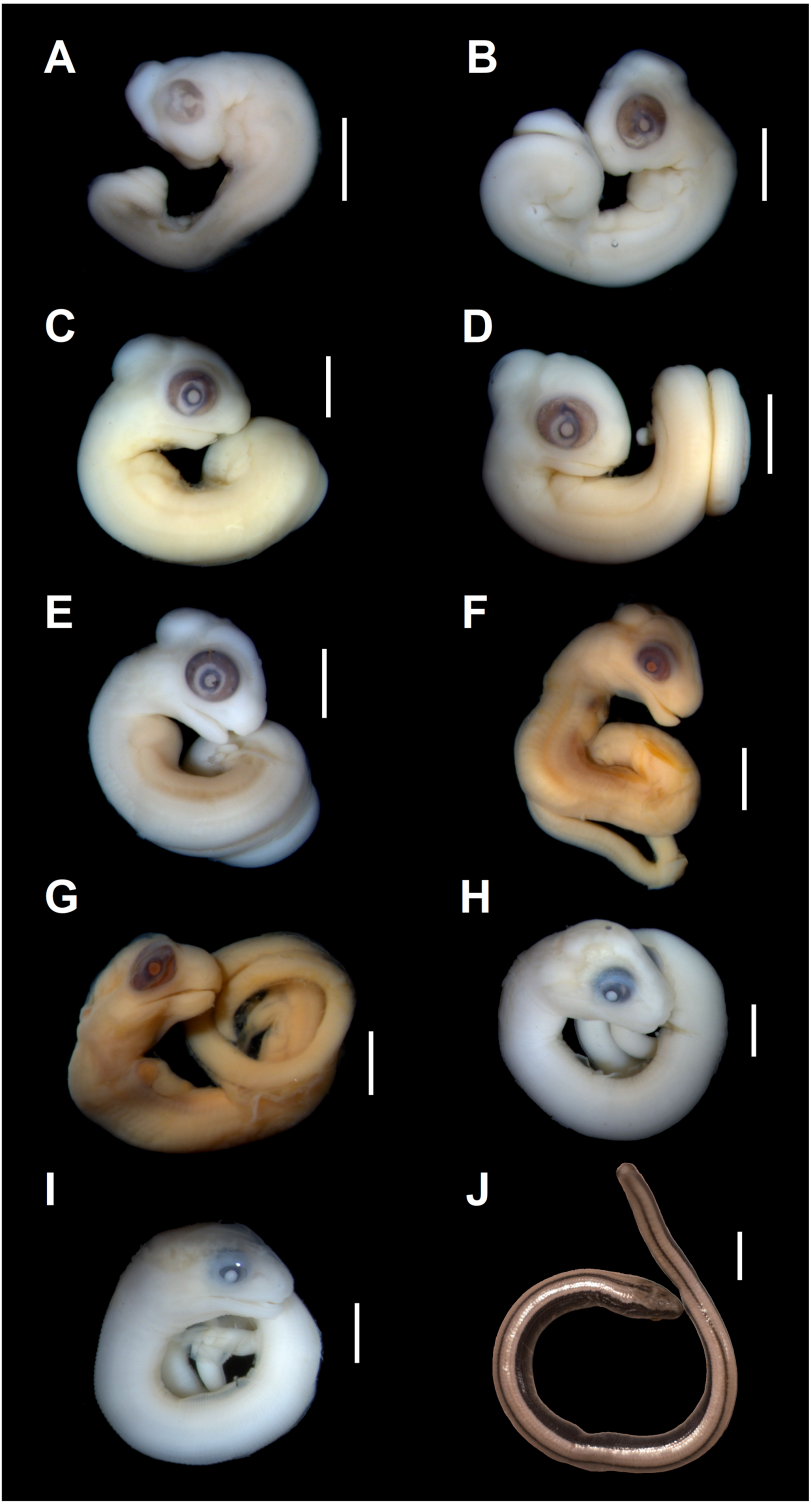
Embryonic development of *Anguis fragilis*. Lateral view of *A. fragilis* embryos at various developmental states. (A) State 1. (B) State 2. (C–E) States 3–4. (F–G) State 5. (H) State 6. (I) State 7. (J) State 8 (perinate). Scale bar = 2 mm (A–I) or 5 mm (J).

**Figure 2:**
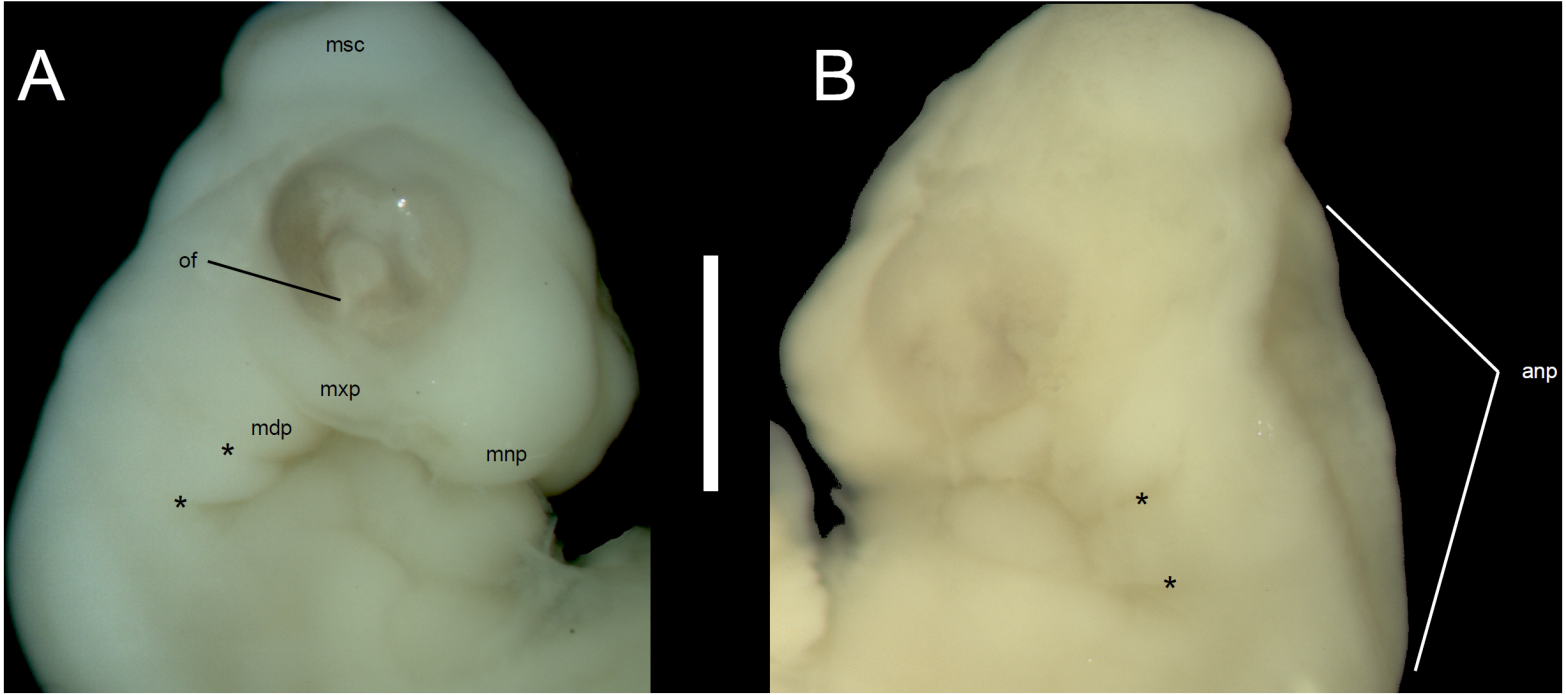
Morphological details of an *Anguis fragilis* embryo. Head of the state 1 embryo in right (A) and left (B) lateral views. Scale bar = 1 mm. Abbreviations: anp, anterior neuropore; mdp, mandibular process; mnp, medial nasal process; msc, mesencephalon; mxp, maxillary process; of, optic (choroid) fissure; *, pharyngeal cleft.

**Developmental state 1**(Fig. 1A)**.** The posterior neuropore is closed but the anterior one is still open. The somites are impossible to distinguish. The mesencephalon forms a bulge at the back of the head, with its apex located just posterior to the mid-point of the eye. The eye is poorly pigmented. The optic (choroid) fissure is open. The medial nasal processes are separated by a deep midline furrow. The anterior end of the maxillary process lies beneath the anterior part of the eye but does not reach the level of its anterior margin. The tip of the mandibular process lies approximately at the midline of the eye. Only two pharyngeal slits can be distinguished. The cervical flexure is about 90° (Figs. 1A, 2).

**Developmental state 2**(Fig. 1B)**.** The entire neural tube is closed. The maxillary process fuses with the frontonasal mass which creates an almost continuous upper jaw. The eye is much better pigmented than in the previous stage and the optic fissure can no longer be observed. The tip of the mandibular process reaches the level of the anterior margin of the eye (Fig. 1B).

**Figure 3:**
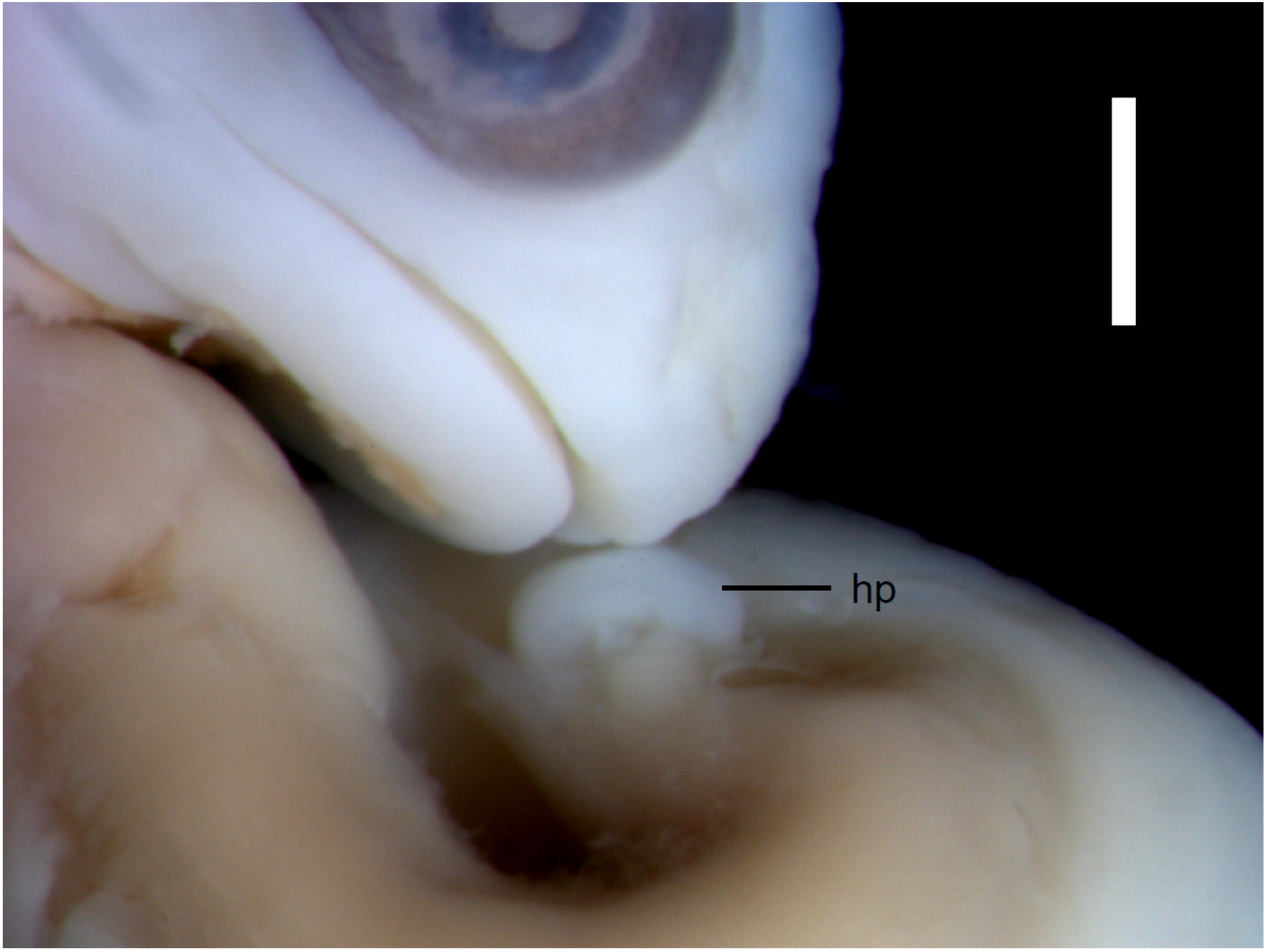
Morphological details of an *Anguis fragilis* embryo. The snout and incipient hemipenis (hp) of the state 3–4 embryo in lateral view. Scale bar = 1 mm.

**Figure 4:**
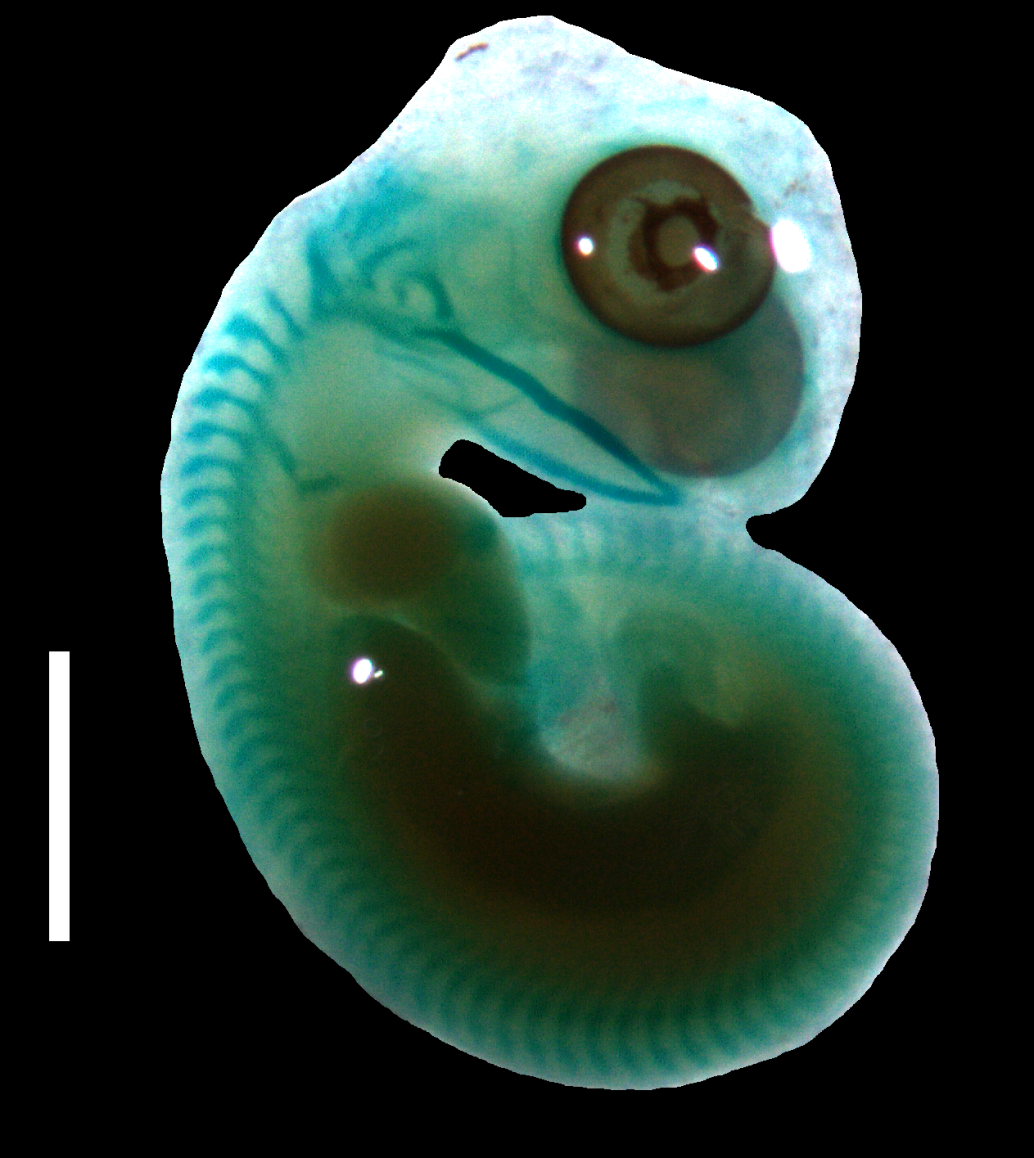
Cartilaginous skeleton of a state 3–4 embryo of *Anguis fragilis*. Scale bar = 2 mm.

**Developmental states 3–4**(Figs. 1C–1E)**.** These stages show the greatest variability in morphological traits. Uniformly, all pharyngeal slits are closed and the pupil and eyelid develop. The entire mesencephalic bulge now lies posterior to the eye. The heart is relatively much smaller than in preceding stages but still protrudes from the body cavity. The hemipenes are present (Fig. 3). The variable traits include: disappearance of the cervical flexure, the length of the snout and the length of the mandible. These features can be present in different combinations, for example, the specimen with no cervical flexure may have a mandible significantly shorter than the upper jaw (reaching approximately the anterior margin of the eye) (Figs. 1C, 1D), while others show the cervical flexure, the mandible already reaching the occlusal point with the upper jaw, but the whole snout is very short (Figs. 1C, 1D). For measurements, see Table S1 and Fig. S1. At these stages, no mineralisations in the skeleton could be detected by double-staining (Fig. 4).

**Developmental state 5**(Figs. 1F, 1G)**.** The body is more elongated and the tail is much less curled than in previous stages. In most specimens the snout is as long or slightly longer than the diameter of the eye. The posterior part of the mandible becomes much deeper. The mesencephalon becomes less prominent but still forms a bulge. In some specimens the external nares become easily observable as depressions (Figs. 1F, 1G).

**Figure 5:**
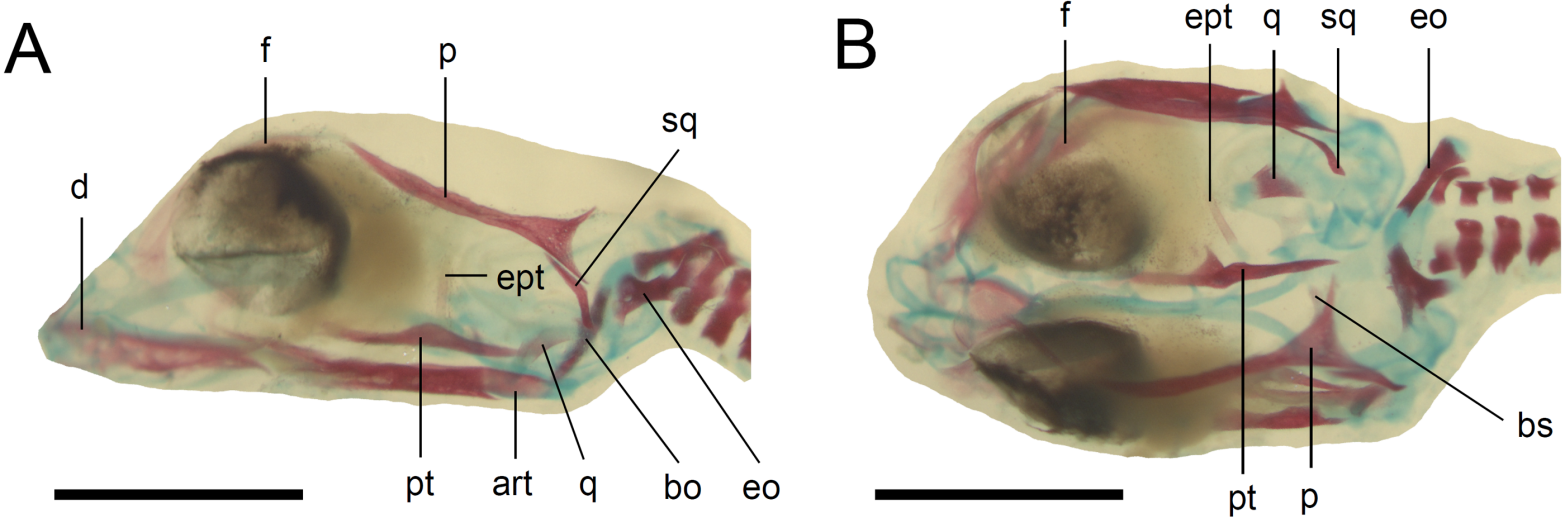
Skull of the double-stained state 6 embryo of *Anguis fragilis*. (A) Lateral view. (B) Dorsal view. Cartilage is stained bluish while bone is stained red. Scale bar = 2 mm. Abbreviations: art, articular; bo, basioccipital; bs, basisphenoid; d, dentary; eo, exoccipital; ept, epipterygoid; f, frontal; p, parietal; pt, pterygoid; q, quadrate; sq, squamosal. Note that the specimen is not fully articulated.

**Figure 6:**
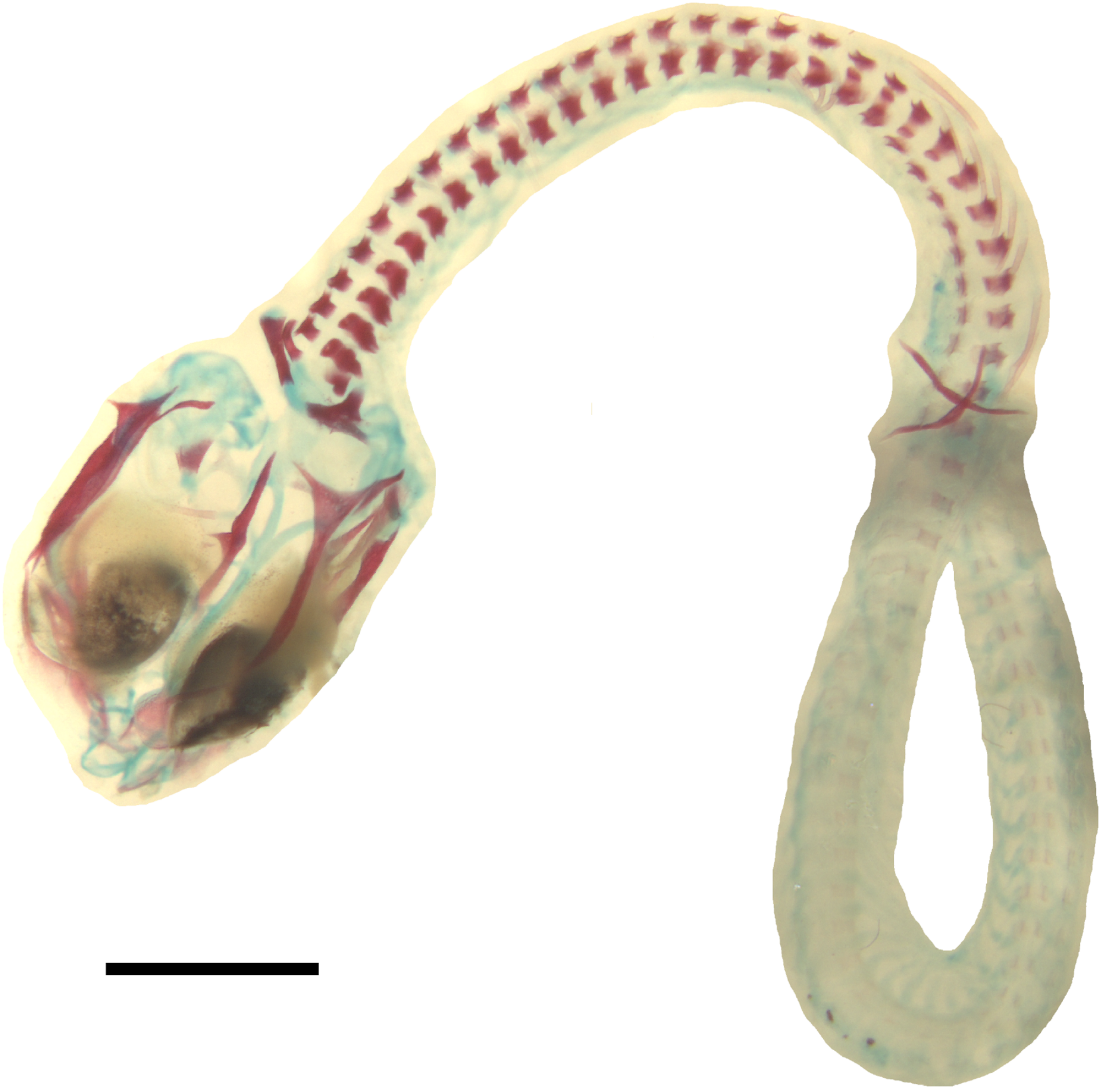
Double-stained state 6 embryo of *Anguis fragilis* in dorsal view. Cartilage is stained bluish while bone is stained red. Note that the cranium is not fully articulated. Scale bar = 2 mm.

**Developmental state 6**(Fig. 1H)**.** The lower eyelid reaches the ventral margin of the pupil. The mesencephalon flattens and no longer takes on a bulge-like form. The head is strongly domed in lateral view. The pineal eye develops on the top of the head, approximately at the level of the posterior margin of the eye. Incipient scales develop on the trunk and venter (Fig. 1H). In the skull, several endochondral bones are ossifying: the exoccipital, basioccipital, basisphenoid, quadrate, epipterygoid and articular. With the exception of the exoccipital and basioccipital they only faintly stain with alizarin. Dermal bones tend to be better ossified. The pterygoid, parietal, frontal, prefrontal, squamosal, maxilla, dentary, angular and supraangular are ossified. The parietals are unfused; only their lateral margins are ossified. The maxilla is present only as a small and faint calcification (Fig. 5). In the vertebral column, the neural arches and vertebral centra are present in the cervical and most of the thoracic vertebrae. The more posteriorly located vertebrae are more poorly ossified or absent (Fig. 6).

**Developmental state 7**(Fig. 1I)**.** In lateral view, the head is much flatter than in the previous stage. The lower eyelid partially covers the ventral part of the pupil. Throat scales develop, while the neck and anterior trunk scales become convex (Fig. 1I).

**Developmental state 8**(Fig. 1J)**.** Perinatal slow worms look almost exactly like adult individuals (Fig. 1J). Main differences concern colouration. Ontogenetic colour changes were described elsewhere (e.g., Juszczyk, 1974).

### Perinatal osteology

**Figure 7:**
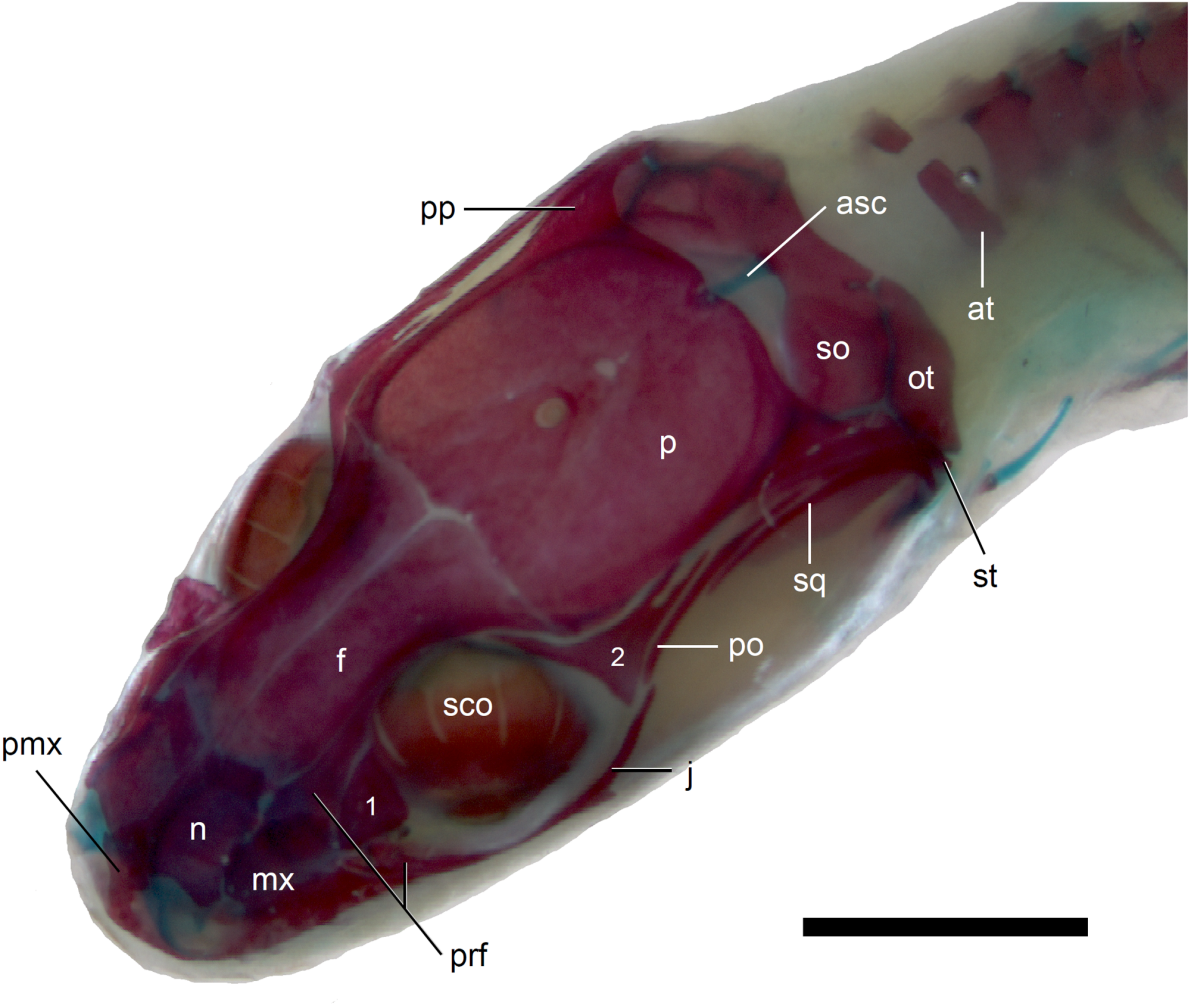
Skull of perinatal *Anguis fragilis* (MNHW-Reptilia-0312) in dorsal view. Scale bar = 2 mm. Abbreviations: asc, ascending process of the supraoccipital; at, atlas; f, frontal; j, jugal; mx, maxilla; n, nasal; ot, otooccipital; p, parietal; pmx, premaxilla; po, postorbital; pp, postparietal process of the parietal; prf, prefrontal; sco, scleral ossicles; so, supraoccipital; sq, squamosal; st, supratemporal; 1, palpebral; 2, postfrontal.

**Figure 8:**
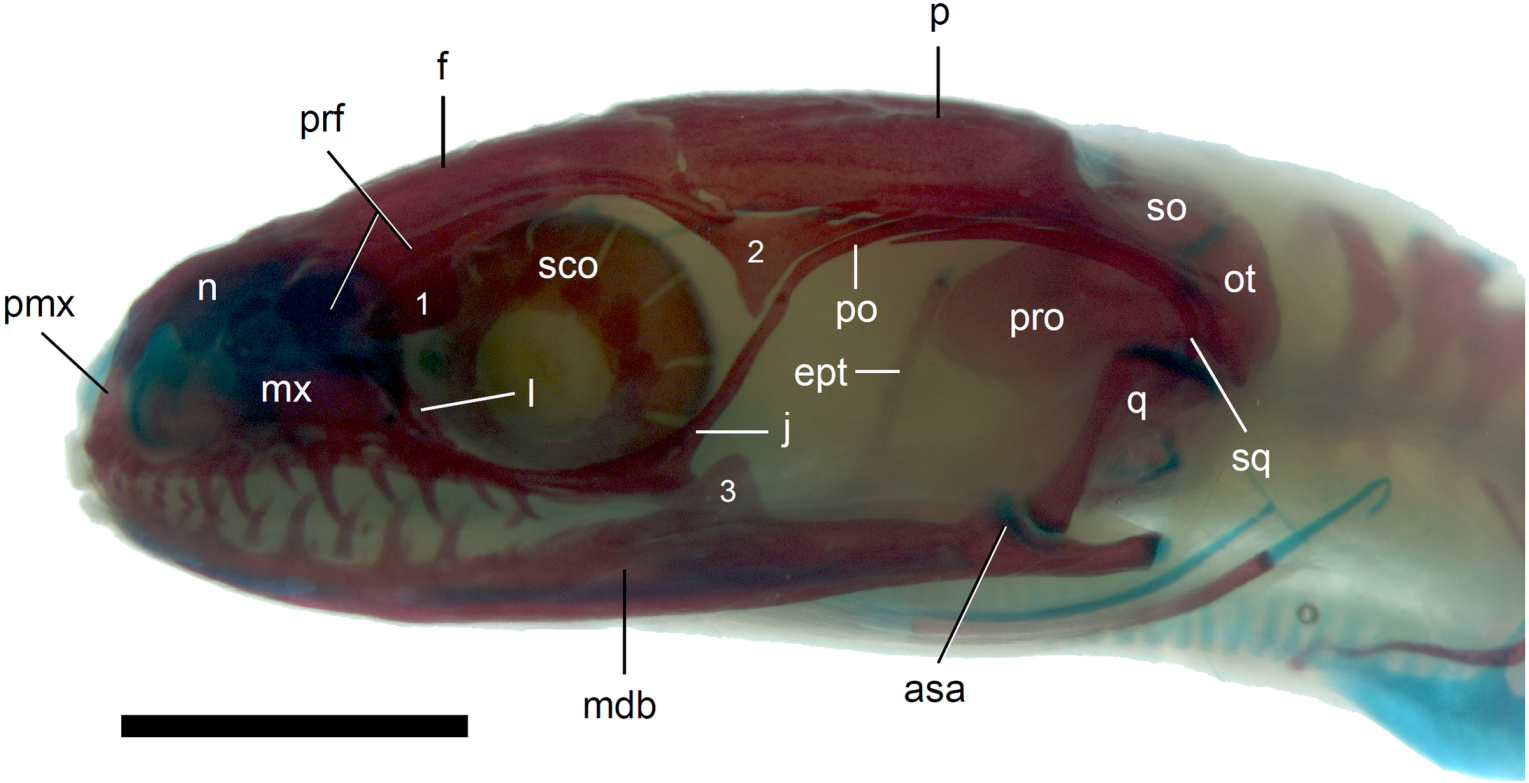
Skull of perinatal *Anguis fragilis* (MNHW-Reptilia-0312) in lateral view. Scale bar = 2 mm. Abbreviations: asa, articular surface of the articular; ept, epipterygoid; f, frontal; j, jugal; l, lacrimal; mdb, mandible; mx, maxilla; n, nasal; ot, otooccipital; p, parietal; pmx, premaxilla; po, postorbital; prf, prefrontal; pro, prootic; q, quadrate; sco, scleral ossicles; so, supraoccipital; sq, squamosal; 1, palpebral; 2, postfrontal; 3, coronoid.

**Figure 9:**
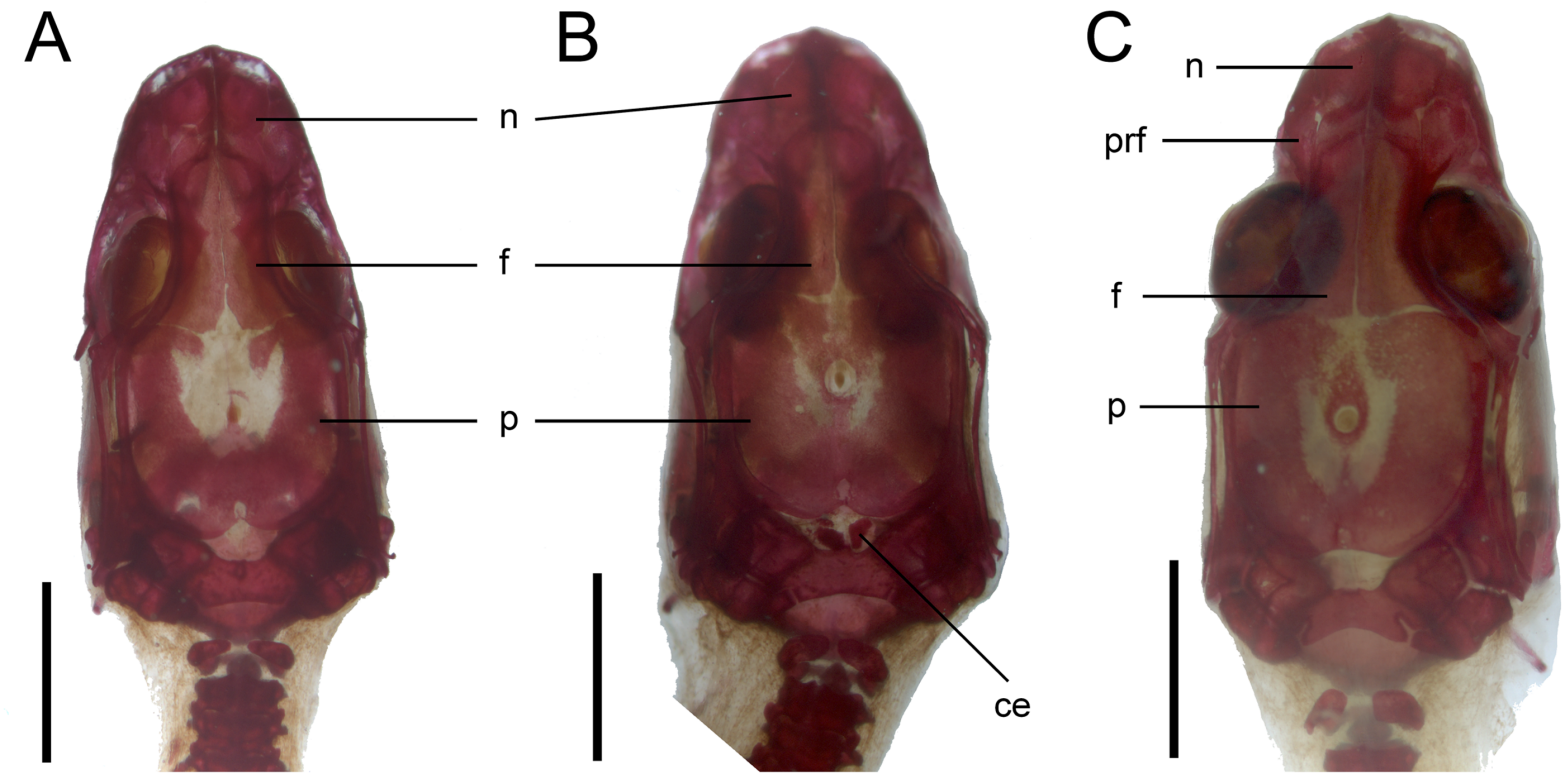
Development of the skull roof in perinatal *Anguis fragilis* in dorsal view. (A) MNHW-Reptilia-0310-4 (SVL = 44 mm), (B) MNHW-Reptilia-0310-6 (SVL = 46 mm), (C) MNHW-Reptilia-0311-1 (SVL = 49 mm). Scale bar = 2 mm. Abbreviations: ce, calcified endolymph; f, frontal; n, nasal; p, parietal, prf, prefrontal.

*Skull*. The ossification of the skull roof is closer to the “less ossified” extreme described by Maisano (2001). The nasal is a paired bone, with an approximately quadrangular shape. Its anterior end is slightly wider mediolaterally than the posterior one. The nasals contact each other medially only in their posterior parts; anteriorly, they are separated by the nasal process of the premaxilla. The nasal contacts the maxilla and prefrontal laterally and the frontal posteriorly (Figs. 7 and 8). The frontal is a paired bone forming a large part of the skull roof. It also creates the dorsal part of the orbit. The frontal is greatly expanded posteriorly, where it meets the parietal. Apart from that, the lateral margins of the bone are approximately straight, there is only a slight narrowing just anterior to the posterior expansion. The frontals are unfused in all studied specimens (Fig. 9) except the largest, in which they began to fuse anteriorly (Fig. 7). In the smallest specimens, the parietal is ossified in its posterior and lateral parts, with the parietal fontanelle being approximately triangular (Fig. 9A). Ossification proceeds also from anterolateral parts towards the midline, along the posterior border of the frontals (Fig. 9B). Additional ossification centres are present around the parietal foramen, from which the ossification spreads anteriorly and posteriorly, so that only parts of the parietal that are located laterally and latero-posteriorly to the parietal foramen remain unossified (Fig. 9C). In the largest specimen, the ossification of the parietal is almost complete, with only a small opening posterior to the parietal foramen still unossified (Fig. 7). The postparietal processes are well- developed and slightly curved ventrally (Fig. 8). The posterior margin is “indented” to form a parietal fossa which contacts the cartilaginous ascending process of the supraoccipital (Fig. 7).

The premaxilla creates the most anterior point of the skull. It presents an alveolar plate, which bears teeth, and an ascending nasal process. In the slow worm, the nasal process slightly narrows dorsal to the alveolar plate, then expands laterally, so that its widest point is just below the nasals in anterior view, and then tapers to a point. The nasal process separates the anterior parts of the nasals (Fig. 7). The maxilla creates most of the lateral part of the snout. It is composed of an anterior, posterior and facial processes. The anterior process creates part of the ventral and posteroventral border of the external naris, while the steep and tall facial process forms its posterior border. Dorsally, the facial process contacts the nasal and prefrontal. Posteriorly, it meets the palpebral and lacrimal. The long and low posterior process contacts the lacrimal dorsally and the jugal posterodorsally, and continues below the orbit (Fig. 8).

**Figure 10:**
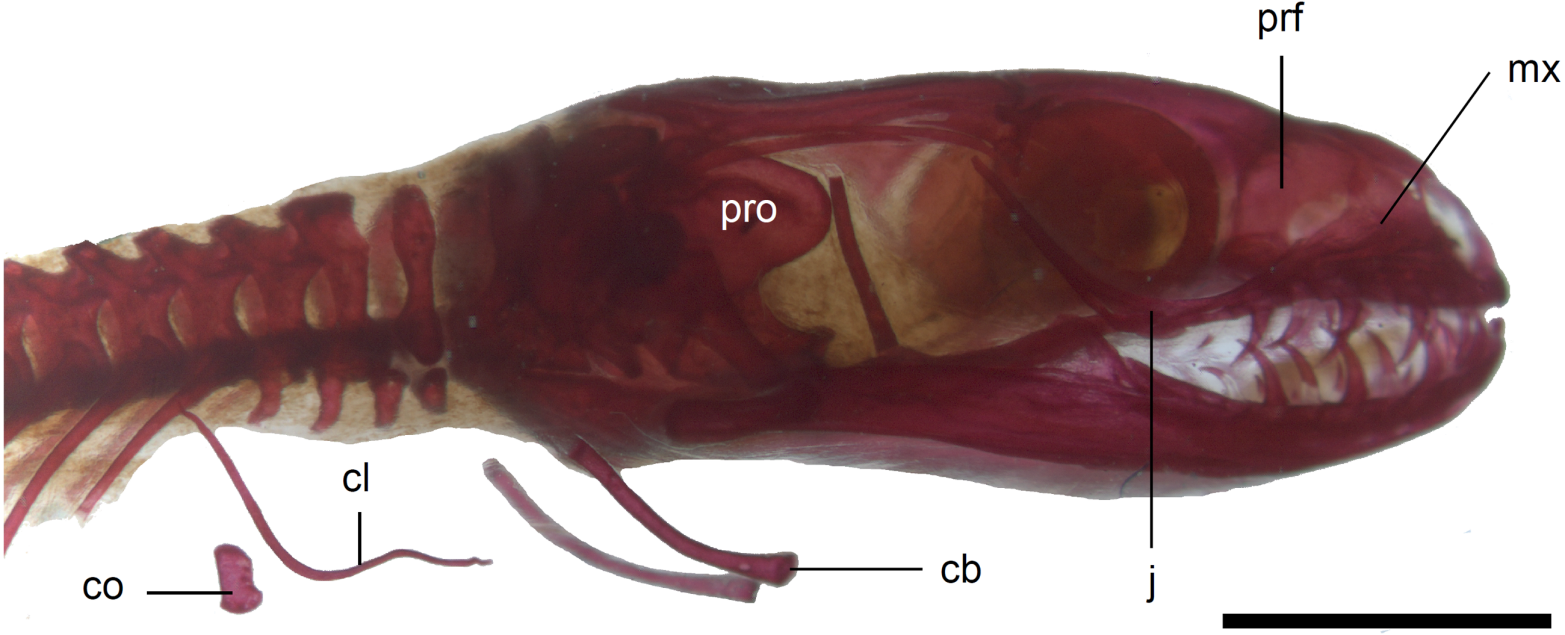
Skull of perinatal *Anguis fragilis* (MNHW-Reptilia-0310-4) in lateral view. Scale bar = 2 mm. Abbreviations: cb, first ceratobranchial; cl, clavicle; co, coracoid; j, jugal; mx, maxilla; prf, prefrontal; pro, prootic.

The prefrontal is composed of the main body and a long and narrow orbital process that extends approximately to the narrowest point of the frontal. The prefrontal contacts the maxilla anteriorly and the nasal anteromedially, and separates the palpebral from the frontal (Fig. 7). In the smallest studied perinates, there is a more-or-less elipsoidal foramen between the posterior margin of the facial process of the maxilla and the anterior margin of the prefrontal (Fig. 10). The palpebral is a relatively large bone, with the shape approximating a right triangle in dorsolateral view. It contacts the jugal and lacrimal ventrally, the maxilla anteriorly and the postfrontal dorsally (Figs. 7, 8). The jugal is a triradiate bone forming most of the ventral and posterior parts of the orbit. It meets the maxilla and lacrimal anteriorly, and the postfrontal and postorbital posterodorsally. The quadratojugal process is present but small (Fig. 8). It is absent in smaller specimens, with SVL between 44 and 46 mm, so it most probably develops postnatally (Fig. 10). The lacrimal creates most of the anterior margin of the orbit. It contacts the maxilla anteriorly and ventrally, jugal posteroventrally and palpebral dorsally. In lateral view, it appears to be separated from the prefrontal by the maxilla-palpebral contact (Fig. 8). The postfrontal is a triradiate bone forming the posterodorsal part of the orbit. The dorsal process is relatively long and slender, contacting the parietal and frontal. The ventral process is shorter and more robust; ventrally, it meets the jugal and excludes the postorbital from the orbit. The posterior process is deeply bifurcated, extending along the parietal and postorbital (Fig. 7). The postorbital is a simply-shaped bone, extending from the jugal and postfrontal anteriorly to the supratemporal posteriorly. Together with the parietal, postfrontal and supratemporal, it creates a small supratemporal fenestra (Fig. 7).

The squamosal is of the typical “hockey-stick” shape but the posterior, ventrally curved part is not strongly expanded. The squamosal runs laterally to the postorbital; it also meets the supratemporal posteriorly (Figs. 7 and 8). The supratemporal is a splint of bone wedged between the postparietal process of the parietal and the squamosal. Like in the latter bone, its posterior part is curved ventrally (Fig. 8).

The quadrate lies lateral to the braincase and together with the articular participates in forming the mandibular joint. The tympanic crest, which extends from the anterodorsal part of the bone to the mandibular condyle, is almost straight. The cephalic condyle is located at the posterodorsal end and almost contacts the squamosal and supratemporal. Dorsally (cephalic condyle) and ventrally (mandibular condyle), the quadrate is still covered by cartilaginous caps (Fig. 8). The epipterygoid is a thin bony rod supported by the pterygoid ventrally and meeting the prootic posterodorsally. In the largest examined specimen, the dorsal end still bears a small cartilaginous cap (Fig. 8).

**Figure 11:**
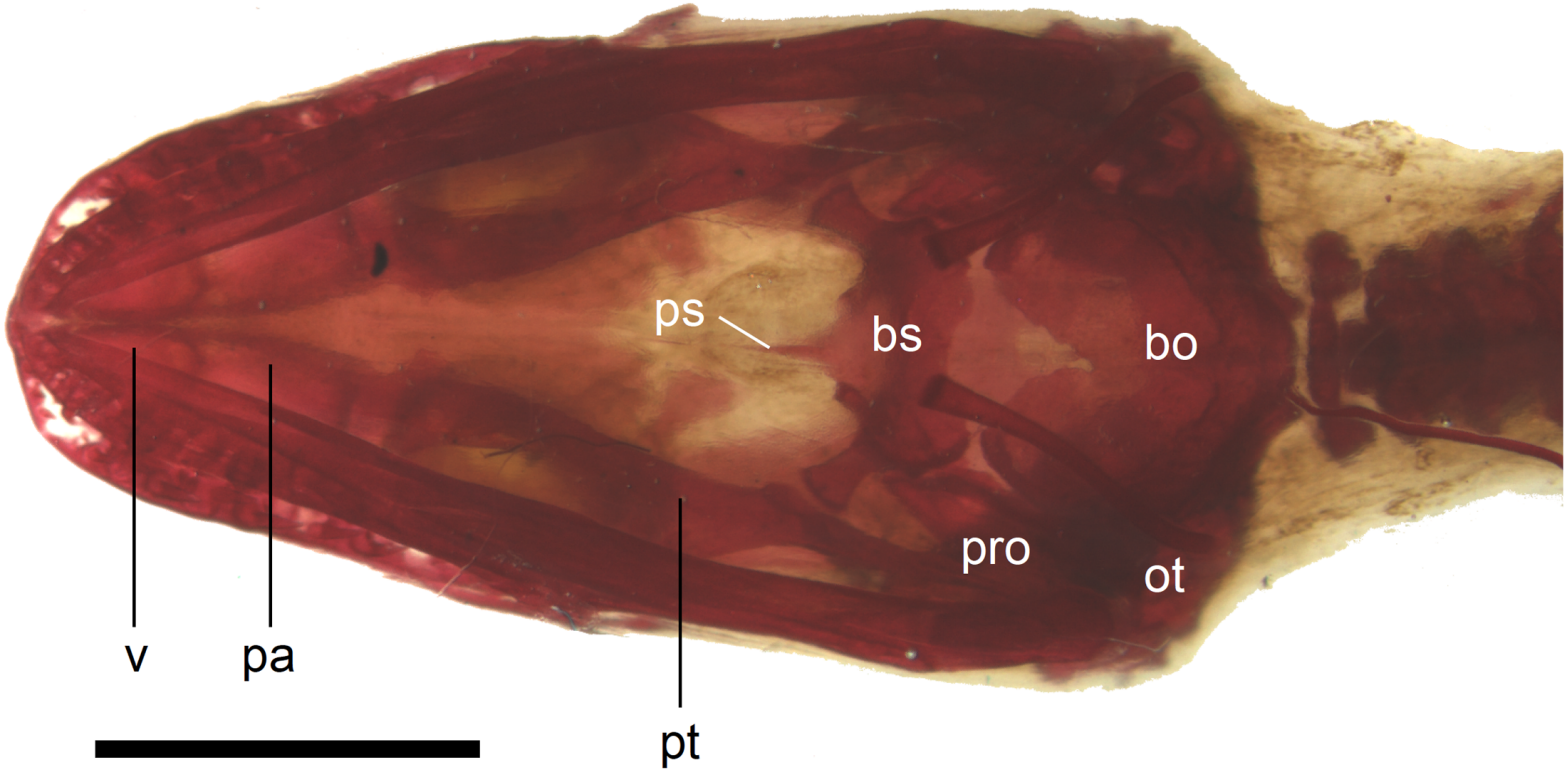
Skull of perinatal *Anguis fragilis* (MNHW-Reptilia-0310-4) in ventral view. Scale bar = 2 mm. Abbreviations: bo, basioccipital; bs, basisphenoid; ot, otooccipital; pa, palatine; pro, prootic; ps, parasphenoid; pt, pterygoid; v, vomer.

In the palate, the pterygoids are widely separated medially and there is no sign of palatal teeth observed. The pterygoid is an approximately y-shaped bone. The posterior process contacts the quadrate laterally. Medially, the main body contacts the basipterygoid process of the parasphenoid. The anterolateral process of the pterygoid meets the ectopterygoid and the long anterior process contacts the palatine with an almost straight suture (Fig. 11). The palatines are widely separated posteriorly, while the space between them is much smaller anteriorly, where they contact the vomers (Fig. 11). The vomers contact each other at the midline (Fig. 11).

**Figure 12:**
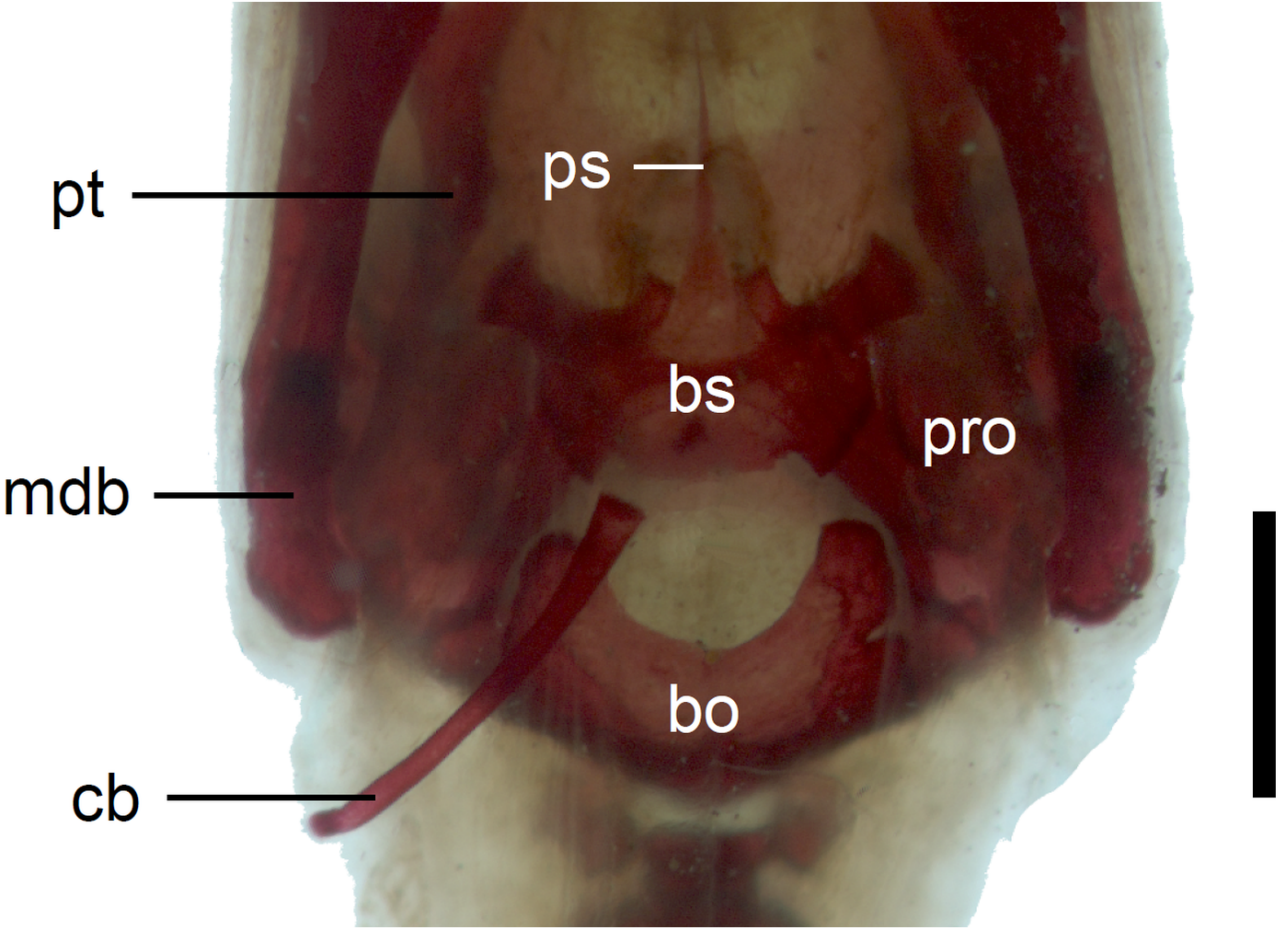
Braincase of perinatal *Anguis fragilis* (MNHW-Reptilia-0311-1) in ventral view. Scale bar = 1 mm. Abbreviations: bo, basioccipital; bs, basisphenoid; cb, first ceratobranchial; mdb, mandible; pro, prootic; ps, parasphenoid; pt, pterygoid.

**Figure 13:**
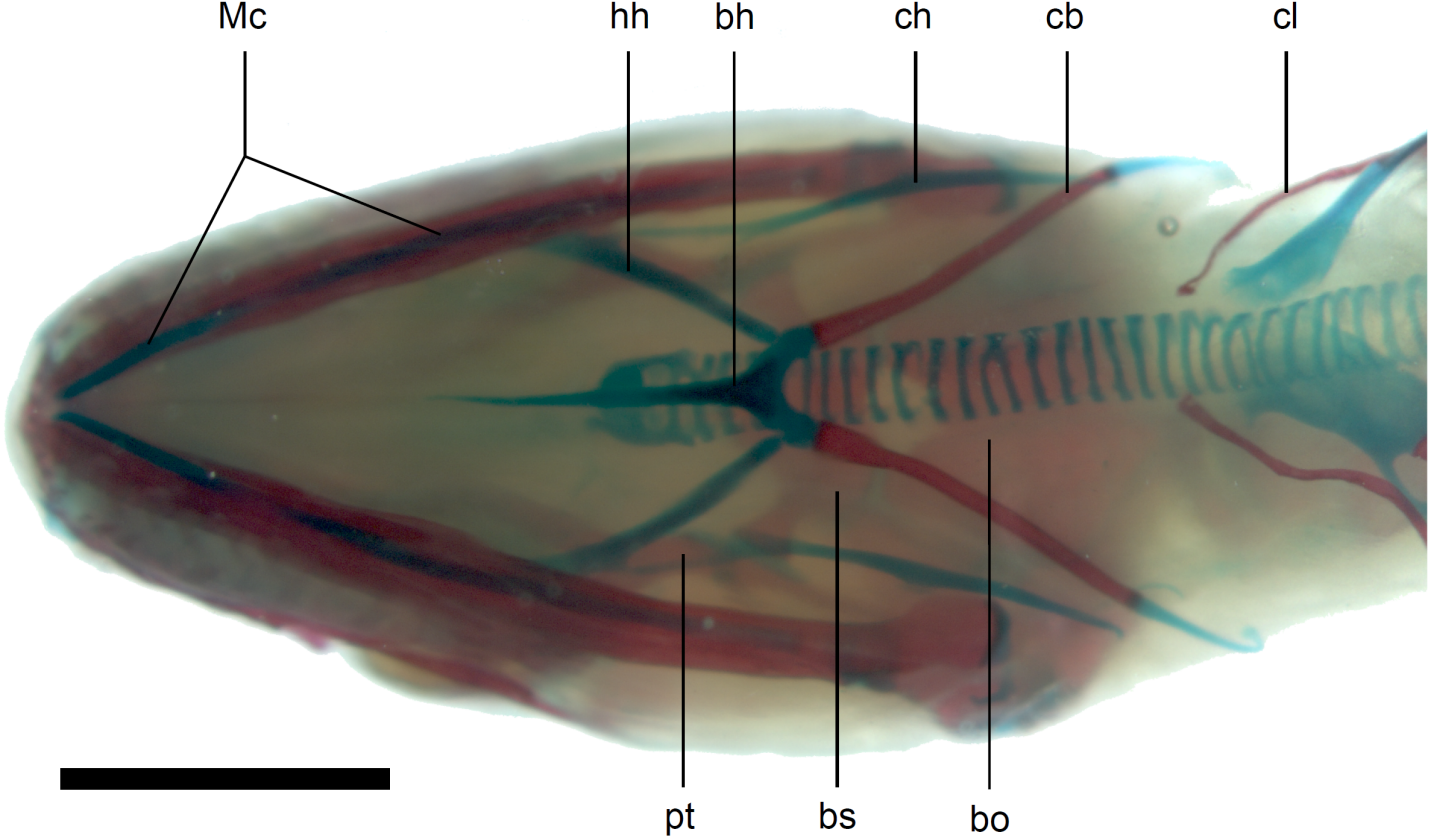
Skull of perinatal *Anguis fragilis* (MNHW-Reptilia-0312) in ventral view. Scale bar = 2 mm. Abbreviations: bh, basihyal; bo, basioccipital; bs, basisphenoid; cb, first ceratobranchial; ch, ceratohyal; cl, clavicle; hh, hypohyal; Mc, Meckel’s cartilage; pt, pterygoid.

In the braincase, only the fusion of the exoccipitals and opisthotics into the otooccipitals takes place prenatally. Other bones remain unfused after birth. Even in the second-largest specimen (MNHW-Reptilia-0311-1, SVL = 49 mm), there are still wide spaces between most of the bones constituting the braincase (Fig. 12). The basicranial fenestra between the basisphenoid and basioccipital persists in the largest neonate examined (Fig. 13). The basioccipital forms the main part of the base of the braincase. In the second-largest specimen, it is approximately U-shaped in ventral view (Fig. 12). Its anterior margin is strongly concave so that the basicranial fenestra is large. Laterally, there is a shallow notch approximately in the mid-length of the bone (Fig. 12). The sphenoid is a composite bone composed of the endochondral basisphenoid and dermal parasphenoid. Both these components are already ossified at the time of birth. The basipterygoid processes have slightly expanded ends which contact the pterygoids in the largest specimen (Fig. 13). These two elements are more widely separated in the second-largest slow worm (Fig. 12). The posterior margin of the basisphenoid is not straight in ventral view but bears two shallow notches, near the left and right margins (Fig. 12). The supraoccipital creates the posterodorsal part of the braincase. Laterally, it contacts the postparietal processes of the parietal (more dorsally) and the prootics (more ventrally). Posterolaterally it also contacts the otooccipitals. The anterior margin of the bone is strongly concave in dorsal view, with anterior tips projecting anteriorly. At the midline, there is a cartilaginous ascending process which extends to the parietal fossa. There are two shallow notches in the posterior margin of the bone, near the dorsolateral borders of the foramen magnum, close to the most median contact with the otooccipitals (Fig. 7). The prootic constitutes the lateral part of the braincase. It contacts the parietal dorsally, the supraoccipital posteromedially, the otooccipital posteriorly and the sphenoid ventrally. It has a relatively large, slightly downturned alar process that meets the dorsal part of the epipterygoid. In the largest studied specimen, it has two small tubercles, both in contact with the epipterygoid; one located near the ‘apex’ of the process and the second, slightly more posterodorsally (Fig. 8). These tubercles were not observed in smaller specimens. The otooccipital forms the posterolateral part of the braincase, as well as the lateral margin of the foramen magnum. In dorsal view, it contacts the prootic anteriorly and the supraoccipital anteromedially. It extends approximately from the posterior end of the supratemporal laterally to the notch in the supraoccipital medially (Fig. 7). The mandible halves are not fused in all studied specimens and Meckel’s cartilage persists even in the largest specimen, in which it extends posteriorly almost to the level of the articular surface for the quadrate. Anteriorly, Meckel’s cartilages are not fused (Fig. 13). The coronoid is not firmly fused to other mandibular bones (Fig. 8) but otherwise, the morphology of the remaining bones (dentary, angular, surangular, articular) is very similar to the adult form. The articular surface for the quadrate, as well as the posterior end of the retroarticular process, are still capped by cartilage (Fig. 8).

Calcified endolymph in dorsal lymphatic sacs within the cranium occurs in only one specimen (Fig. 9B). The scleral ossicles were ossified in all studied specimens (Figs. 8 and 10). The ossification of the first ceratobranchial was also completed prenatally. In the largest specimen, the basihyal and hypohyals are calcified (Fig. 13). No calcifications in the trachea or larynx were observed.**

**Figure 14:**
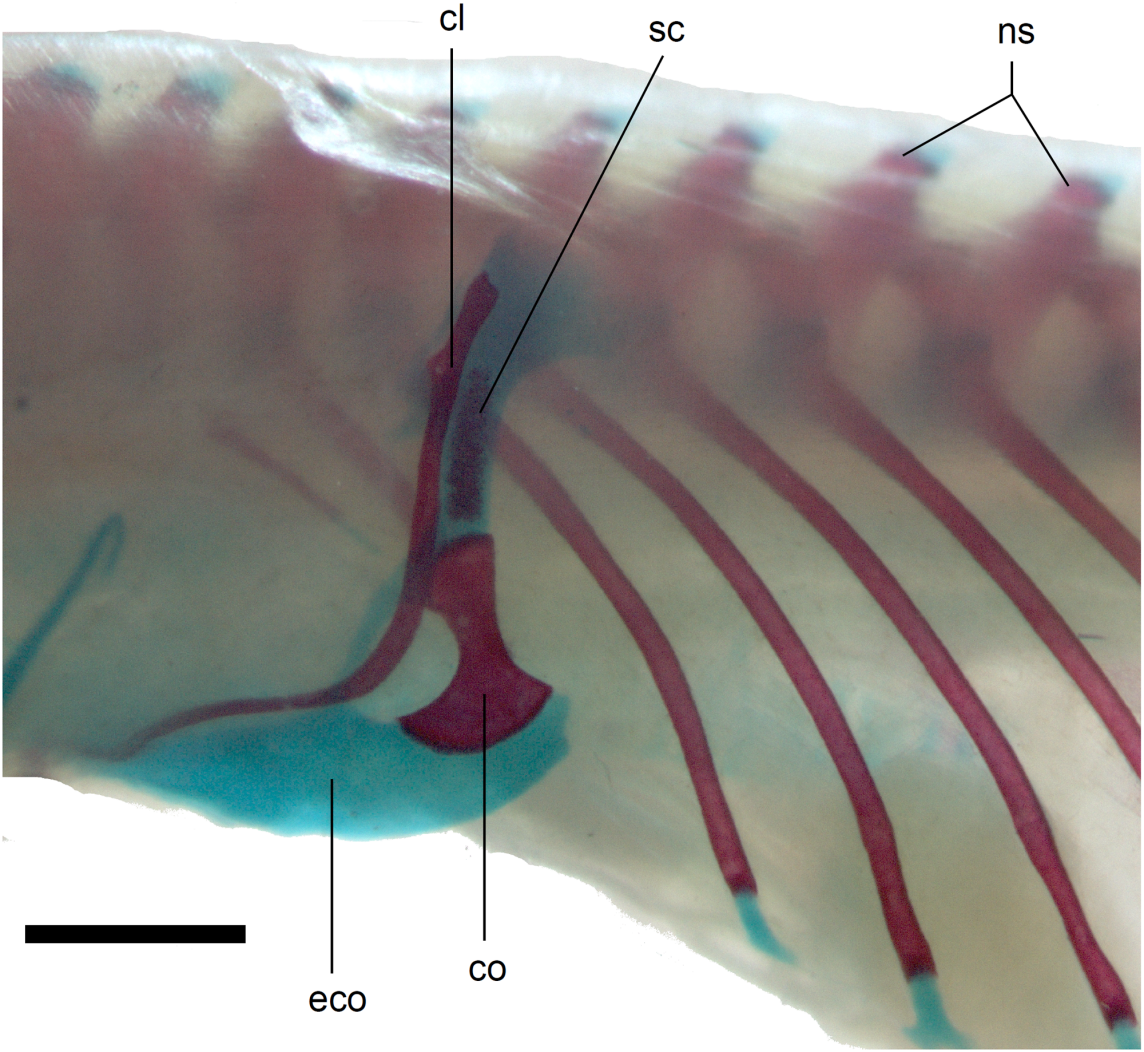
Pectoral girdle of perinatal *Anguis fragilis* (MNHW-Reptilia-0312) in lateral view. Scale bar = 2 mm. Abbreviations: cl, clavicle; co, coracoid; eco, epicoracoid cartilage; ns, neural spine; sc, scapula.

*Vertebral column*. The ossification of the vertebral column is relatively advanced near the time of birth. Both neural arches are already fused in postatlantal vertebrae of the smallest studied slow worms (MNHW-Reptilia-0310-4 and 0310-5, both with SVL = 44 mm). Similarly, the neurocentral sutures of all vertebrae except the atlas are closed. In the atlas, both neural arches and the centrum remain separate in all studied specimens (Figs. 7 and 9). Low neural spines are present even in the smallest specimens and their ossification is almost complete in the largest one, with only the tips still being cartilaginous (Fig. 14). The sacral ribs do not contact one another in any of the studied slow worms.

**Figure 15:**
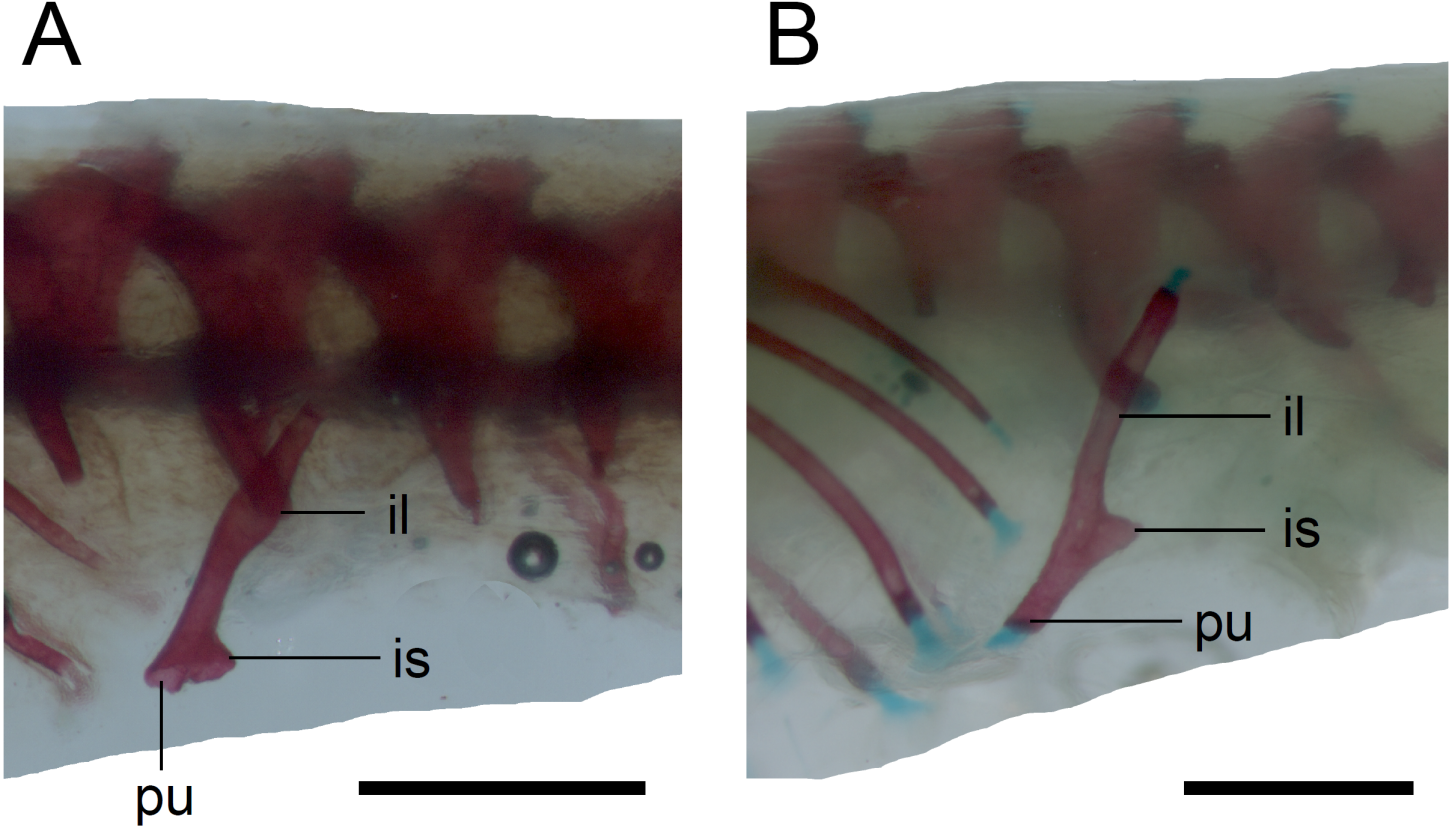
Pelvic girdle in perinatal *Anguis fragilis* in lateral view. (A) MNHW-Reptilia-0311-1 (SVL = 49 mm). (B) MNHW-Reptilia-0312 (SVL = 55 mm). Scale bar = 1 mm. Abbreviations: il, iliac process; is, ischiac process; pu, pubic process.

*Limb girdles*. In the pectoral girdle, only the coracoid is ossified in all but the largest studied specimen. It is a small bone with expanded dorsal and especially ventral ends (Fig. 10). In the largest specimen, the expansions further increase. Additionally, a small scapula ossifies dorsal to the coracoid. There is no sign of fusion between these two bones (Fig. 14). In the pelvic girdle of all perinatal specimens, all three pelvic bones could be distinguished which indicates that even the very small pubis and ischium ossify prenatally. The pubis is represented by an anteriorly projecting pubic process, with its anterior end capped by cartilage (although the cartilage was stained only in the largest specimen), and the ischium is present as a posteriorly projecting ischiac process (Figs. 15A, 15B). However, even in the second largest lizard, these two processes are still very small (Fig. 15A).

## Discussion

It is difficult to directly compare the development of *Anguis fragilis* to other anguimorphs because several stages are missing in the sequence of the former species. Although the available data (Gregorovicova et al., 2012; Werneburg, Polachowski & Hutchinson, 2015; Andrews et al., 2017) suggest that the developmental sequences are similar, one of the apparent differences is the timing of appearance of the external nares. In *Varanus indicus* (Gregorovicova et al., 2012) and especially in *V. panoptes* (Werneburg, Polachowski & Hutchinson, 2015), they could be observed at relatively much earlier stages than in *A. fragilis*. The nasal region of the chondrocranium shows a paedomorphic state in *Anguis* (Yaryhin et al., 2021) but it is unclear whether these two features could be linked. Almost certainly it is an artefact of preservation, because the external nares in squamates generally, develop at earlier stages than was observed in our sample of *A. fragilis* (e.g., Kaczmarek, Metscher & Rupik, 2021). In addition, newly-formed external nares are filled by a nasal plug (e.g., Kaczmarek, Metscher & Rupik, 2021) which further hinders their identification.

Interestingly, in a few clutches, we observed high variation in characters considered important in developmental tables, such as the disappearance of cervical flexure or mandible length. Therefore, some embryos dissected from a female would be classified in a different stage from some of their siblings (for example, lizards shown in Figs. 1C and 1D were dissected from the same female). A similar situation was described in a typhlopid snake *Amerotyphlops brongersmianus* (Sandoval, García & Álvarez, 2020). In that case, the authors suggest that this variation may result from asynchronous development during the early phase of embryonic development when the effect of external (environmental) factors is the greatest (Sandoval, García & Álvarez, 2020). Indeed, some characters occurring in the early developmental phase were shown to be quite variable in the iguanian *Uta stansburiana* (Andrews & Greene, 2011). Many factors affecting embryonic development have been identified in lizards. Light can significantly accelerate embryonic development (Zhang et al., 2016). However, in viviparous species such as *A. fragilis* the embryos develop within the body cavity of the female. This also would not explain the variation within a single clutch. Temperature is another factor because higher temperatures usually accelerate development (Noble, Stenhouse & Schwanz, 2018). The temperature within the animal body is not identical in different parts (e.g., Schmidt-Nielsen, 1997), so it seems possible that embryos developing closest to heat-producing organs such as heart or stomach would be in a slightly more advanced stage of development when compared to embryos developing in slightly cooler places. Unfortunately, information about the topographic position of given embryos within the female body is not always available. Also, in most of the clutches, the morphological differences between embryos are minimal. Thus, the effect of temperature on this variation, if any, seems to be very limited. Embryonic development may also be affected by the availability of oxygen in the oviducts, which is smaller in larger clutches (Foucart, Heulin & Lourdais, 2017), but our data are too limited to tell whether this may be the case in *A. fragilis*. Importantly, we observed the variation in later stages than was reported in *Am. brongersmianus* (Sandoval, García & Álvarez, 2020), when the effect of the environment is presumed to be smaller. Therefore, it cannot be excluded that some intrinsic, genetic factors could play a role. One of the potential explanations is multiple paternity which affects the time of fertilisation and, therefore, the age of the embryos. Such small differences in age could be reflected in the morphology of the embryos. Although multiple paternity has not yet been reported for *A. fragilis*, this phenomenon is known to occur widely in reptiles (Uller & Olsson, 2008). Additionally, multiple paternity is usually present only in a fraction of the clutches in a given population or species (Uller & Olsson, 2008). This would be consistent with the fact that only some clutches of *A. fragilis* showed such phenotypic variation. We aim to test this hypothesis in a forthcoming contribution.

The degree of calcification in the vertebral column in a late embryo (developmental state 6) indicates an anteroposterior order of ossification in the postcranial axial skeleton. This pattern is predominant in squamates (e.g., Hugi et al., 2012; Roscito & Rodrigues, 2012), however, in another anguimorph, *Varanus panoptes*, all vertebrae seem to ossify nearly simultaneously, without a clear gradient (Werneburg, Polachowski & Hutchinson, 2015). The dermatocranium develops earlier than the neurocranium in *A. fragilis*, an almost universal feature in squamates (Evans, 2008). This state 6 embryo seems to be at an earlier developmental stage than the one illustrated by Leydig (1872), as indicated by the lack of the ossified palatine, premaxilla and vomer. Interestingly, we observed only a small and faint ossification in the maxilla (while the other tooth-bearing bone of the upper jaw, the premaxilla, is absent). Thus, the maxilla seems to ossify relatively later in *A. fragilis* than it does in other studied anguimorphs, *Elgaria coerulea* and *V. panoptes* (Good, 1995; Werneburg, Polachowski & Hutchinson, 2015). On the other hand, the ossification of the articular is accelerated in *A. fragilis* in comparison to *E. coerulea*, in which it is the last cranial bone to ossify (Good, 1995).

Several aspects of the skeletal anatomy of *A. fragilis* were controversial for many years. For example, the episternum (interclavicle) was described as being variably present in slow worms (Fürbringer, 1870; Krieg, 1919), while Stokely (1947) failed to detect any such ossification. Our observations agree with those of Stokely—in none of the individuals in our sample, either embryonic, perinatal or adult, was this bone present. As Stokely (1947), we also did not observe any sign of the sternum—either cartilaginous or ossified—in *A. fragilis*.

The skull of the perinatal slow worms is relatively poorly ossified. None of the braincase bone fusions listed by Maisano (2001) was observed in *A. fragilis*. This is similar to the viviparous gerrhonotine *Elgaria coerulea* which is also characterised by a poorly fused neonatal skull (Maisano, 2001). However, *A. fragilis* shows better ossified parietals because even in the smallest specimens in our sample the parietals were already fused posteriorly and some parts of the parietal table were ossified as well, in contrast to *E. coerulea* in which only lateral margins were ossified (Maisano, 2001). The pattern of parietal ossification also seems to be different between these two species. In *A. fragilis* the centrally-located parts of the bone (laterally and posterolaterally to the parietal foramen) are the last to ossify, while in *E. coerulea* the most anterior part of the bone (which contacts the frontals) is the last (Maisano, 2001). Maisano (2001) hypothesised that such poor ossification of the neonatal skeleton in *E. coerulea* may be related to the viviparity of this lizard because viviparous lizards tend to have more poorly ossified skeletons at the time of birth than do oviparous ones. Data from *A. fragilis* are consistent with this pattern. Additionally, a late embryo of an oviparous monitor lizard, *Varanus panoptes*, has a much better-ossified skull than do both *E. coerulea* and *A. fragilis*. In *V. panoptes*, the ossification of the frontals and parietal is almost complete prenatally, while in the braincase, at least the basioccipital and otooccipitals seem to be fused, based on published figures (Werneburg, Polachowski & Hutchinson, 2015). On the other hand, the frontoparietal region of a late embryo of a viviparous diploglossine (or diploglossid) *Celestus costatus* is better ossified than in neonates of *A. fragilis* or *E. coerulea* (Da Silva et al., 2018). However, the latter two species are more closely related to each other than to *C. costatus* (e.g., Wiens & Slingluff, 2001; Conrad et al., 2011), so the effect of phylogeny also cannot be ruled out. Therefore, a link between viviparity and skeletally immature neonates is not unambiguous and still more data are needed. Interestingly, the vertebral column of *A. fragilis* is well-developed in comparison to most other studied squamates (Maisano, 2001). The neurocentral suture of all vertebrae except the atlas is closed, the neural arches are fused and the neural spines are ossified. In an extensive study, all these three characters were present in neonates only in the amphisbaenian *Bipes biporus*, which has robust forelimbs but lacks hindlimbs (Maisano, 2001). Obviously, in limbless or almost limbless squamates, the vertebral column is the most important part of the skeletal system that plays a role in locomotion (e.g., Gans, 1962). Because neonates must be able to move immediately after hatching or birth, it seems logical that the vertebral column would be more strongly ossified in limbless taxa than in species with well-developed limbs. This is consistent with the observation that in skinks with reduced limbs the cervical vertebrae ossify earlier (Hugi et al., 2012). However, this conclusion must remain tentative because the number of studied squamate species (especially limbless ones) is still not sufficient (unfortunately, descriptions of the vertebral column in late embryos of two gymnophthalmids with strongly-reduced limbs, *Calyptommatus sinebrachiatus* and *Nothobachia ablephara*, are not explicit enough to enable more detailed comparisons with *A. fragilis*; Roscito & Rodrigues, 2012). Regardless of these problems, it seems that the state of ossification of the neonatal skeleton in squamates is a result of trade-offs between numerous variables such as phylogeny, ovi- or viviparity, and potentially limblessness.

## Conclusions

We described several stages of embryonic development in *Anguis fragilis* which represent early, middle and late developmental phases. This represents a starting point for future studies on the development of this species, especially with more densely-sampled late developmental phases. Interestingly, within a few clutches, we observed a high variation in morphological characters which are often considered important in the classification of embryos into stages. The causes of this variation remain unknown but one of the potential explanations may be multiple paternity which affects the time of fertilisation and thus may cause slight morphological differences between individuals from the same clutch.

The perinatal cranium is relatively poorly ossified. No fusions in the braincase could be observed even in the largest examined lizard. The frontals are unfused (only in the largest specimen have they started to fuse anteriorly), and the parietal is ossified mostly in lateral and posterior parts. However, the state of ossification seems to be at a more advanced stage than in another viviparous anguid, *Elgaria coerulea*. In contrast to the skull, the vertebral column is well ossified at the time of birth which may be related to the greater importance of the spine in locomotion in limbless species. The state of ossification at the time of hatching or birth in squamates is probably affected by trade-offs between numerous factors, including phylogenetic position, mode of reproduction and, potentially, limblessness. However, studies on a greater number of species are necessary to gain better understanding of the importance of these variables in different clades.

##  Supplemental Information

10.7717/peerj.11621/supp-1 Supplemental Information 1 Morphometric data of the studied *Anguis fragilis* embryos The specimens were measured digitally using images. Because the embryos are very fragile and most of them are strongly curled, it was impossible to measure, for example, the snout-vent-length. Definitions of the metric characters are shown in Fig. S1. We did not measure a given distance if the deformation of the specimen was apparent. However, deformations are often difficult to recognise, so the measurements given below should be taken with caution.

10.7717/peerj.11621/supp-2 Supplemental Information 2 Measurements of *Anguis fragilis* embryos ED, eye diameter; HL, head length; MDL, mandible length; SL, snout length; UJL, upper jaw length.

## Additional Information and Declarations

### Data Availability

The following information was supplied regarding data availability: All specimens described in this article or used as a comparative material are deposited in the Museum of Natural History, University of Wrocław (specimen numbers: MNHW-Reptilia-280, MNHW-Reptilia-0310-4, MNHW-Reptilia-0310-5, MNHW-Reptilia-0310-6, MNHW-Reptilia-0311-1, MNHW-Reptilia-0312) or in the Department of Evolutionary Biology and Conservation of Vertebrates, University of Wrocław (IZK 01004, IZK 01005, IZK 01006).
