# Supplementary material for: Embryonic development and perinatal skeleton in a limbless, viviparous lizard, Anguis fragilis (Squamata: Anguimorpha)
Source: PeerJ. 2021 Jun 17;9:e11621. doi: 10.7717/peerj.11621 (PMC8214852; doi:10.7717/peerj.11621)
Supplement: Supplemental Information 1 — The specimens were measured digitally using images. Because the embryos are very fragile and most of them are strongly curled, it was impossible to measure, for example, the snout-vent-length. Definitions of the metric characters are shown in Fig. S1. We did not measure a given distance if the deformation of the specimen was apparent. However, deformations are often difficult to recognise, so the measurements given below should be taken with caution. [file peerj-09-11621-s001.docx]

| **Female (catalogue number)** | **Specimen code** | **Head length** | **Upper jaw length** | **Mandible length** | **Snout length** | **Eye diameter** |
| --- | --- | --- | --- | --- | --- | --- |
| MNHW-Reptilia-0316-3 | S01O02 | Not measured prior to double-staining | | | | |
|  | S01O03 | 3.94 mm | 2.04 mm | 1.77 mm | 1.27 mm | 1.67 mm |
|  | S01O04 | 4.27 mm | 2.48 mm | 2.14 mm | 1.14 mm | 1.63 mm |
|  | S01O05 | Not measured prior to double-staining | | | | |
| MNHW-Reptilia-0315-4 | S06O04 | 3.13 mm | Deformed | Deformed | 0.72 mm | 1.40 mm |
|  | S06O07 | 2.87 mm | Not possible to measure accurately | | 0.80 mm | 1.38 mm |
| MNHW-Reptilia-0315-5 | S07O01 | Damaged specimen | | | | |
|  | S07O02 | Damaged specimen | | | | |
|  | S07O03 | Damaged specimen | | | | |
|  | S07O04 | 2.48 mm | No continuous upper jaw | | 0.67 mm | 0.96 mm |
|  | S07O05 | 2.33 mm | No continuous upper jaw | | 0.84 mm | 0.98 mm |
|  | S07O06 | Damaged specimen | | | | |
| MNHW-Reptilia-0251 | S09O01 | 3.75 mm | 1.92 mm | 1.64 mm | 0.85 mm | 1.45 mm |
|  | S09O02 | Deformed | Deformed | Deformed | Deformed | 1.61 mm |
|  | S09O03 | 4.22 mm | 2.15 mm | 1.73 mm | 1.11 mm | 1.50 mm |
|  | S09O04 | 3.97 mm | 1.74 mm | 1.70 mm | 0.81 mm | 1.57 mm |
|  | S09O05 | 3.80 mm | 2.02 mm | 1.95 mm | 1.12 mm | 1.68 mm |
|  | S09O06 | 3.89 mm | Deformed | 1.62 mm | 0.89 mm | 1.58 mm |
|  | S09O08 | Destroyed specimen | | | | |
|  | S09O09 | 3.93 mm | 2.01 mm | 1.91 mm | 1.40 mm | 1.53 mm |
| IZK 01007 | S10O01 | Deformed specimen | | | | |
|  | S10O02 | Deformed specimen | | | | |
|  | S10O03 | Deformed | 2.38 mm | 2.26 mm | 1.34 mm | Deformed |
|  | S10O04 | Deformed specimen | | | | |
|  | S10O05 | 3.98 mm | 1.88 mm | 1.78 mm | 1.00 mm | 1.28 mm |
|  | S10O06 | Destroyed specimen | | | | |
|  | S10O07 | Deformed | 2.08 mm | 2.03 mm | 1.25 mm | 1.46 mm |
|  | S10O08 | 3.98 mm | 2.01 mm | 1.94 mm | 1.33 mm | 1.28 mm |
| IZK 01008 | S11O01 | Deformed | Deformed | Deformed | Deformed | 1.21 mm |
|  | S11O02 | 3.73 mm | 2.26 mm | 2.25 mm | 1.29 mm | 1.41 mm |
|  | S11O03 | 3.70 mm | 2.40 mm | 2.35 mm | 1.36 mm | 1.40 mm |
|  | S11O04 | 3.87 mm | 2.41 mm | 2.33 mm | 1.16 mm | 1.49 mm |
|  | S11O05 | Probably slightly deformed specimen | | | | |
|  | S11O06 | Deformed | Deformed | Deformed | Deformed | Deformed |
|  | S11O07 | Probably slightly deformed specimen | | | | |
|  | S11O08 | Probably slightly deformed specimen | | | | |
|  | S11O09 | 3.60 mm | Deformed | Deformed | 1.40 mm | 1.46 mm |
|  | S11O10 | Deformed specimen | | | | |
|  | S11O11 | 4.06 mm | 1.97 mm | 1.81 mm | 1.11 mm | 1.57 mm |
| Unknown | S12O01 | 6.06 mm | 3.39 mm | 3.25 mm | 1.65 mm | Partially covered by eyelids |
| Unknown | S12O02 | 5.27 mm | 2.84 mm | 2.82 mm | 1.36 mm | Partially covered by eyelids |
| Unknown | S12O03 | 5.49 mm | 3.37 mm | 3.36 mm | 1.38 mm | Partially covered by eyelids |
