# Supplementary figures and images for: Embryonic development and perinatal skeleton in a limbless, viviparous lizard, Anguis fragilis (Squamata: Anguimorpha)
Source: PeerJ. 2021 Jun 17;9:e11621. doi: 10.7717/peerj.11621 (PMC8214852; doi:10.7717/peerj.11621)

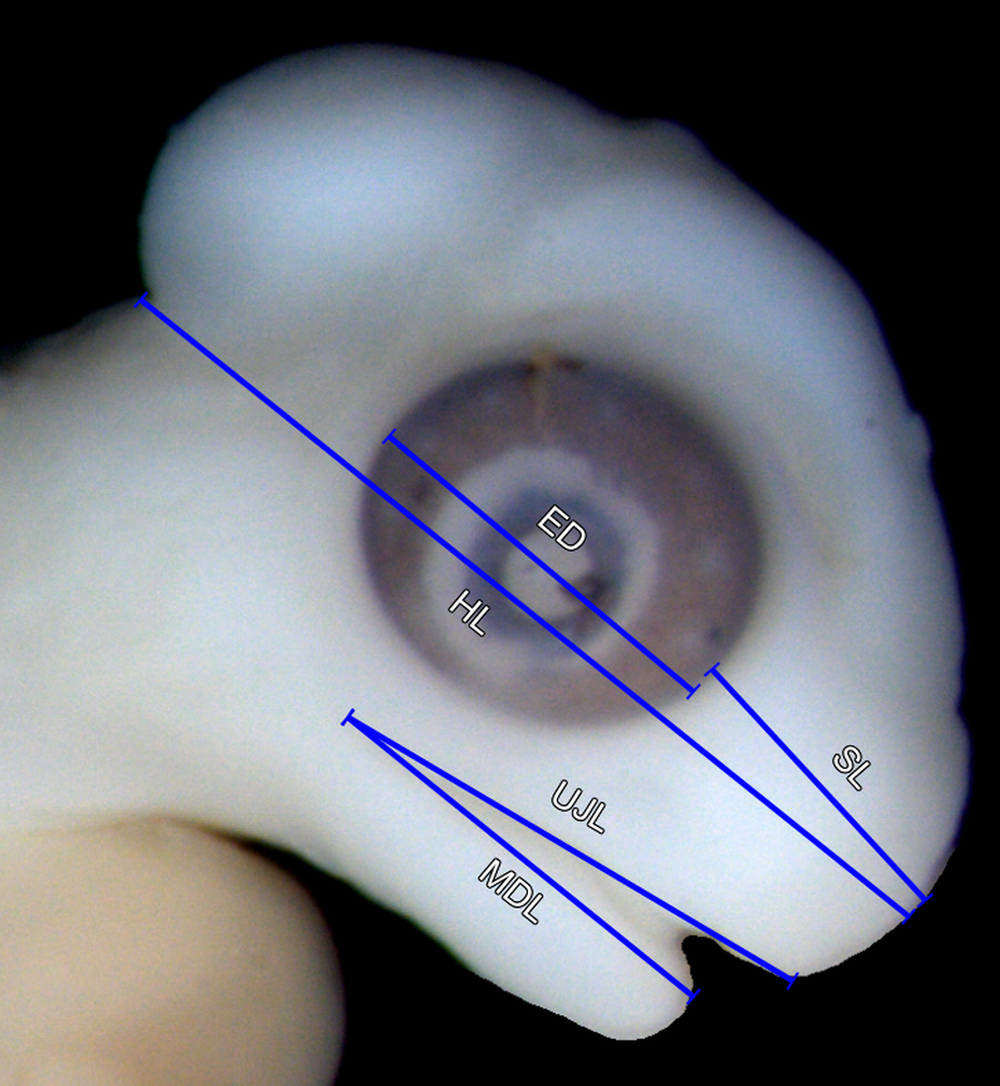

Supplement: Supplemental Information 2 — ED, eye diameter; HL, head length; MDL, mandible length; SL, snout length; UJL, upper jaw length. [file peerj-09-11621-s002.png]
